# On Certain Cases of Intestinal Obstruction

**Published:** 1856-02

**Authors:** 


### ECLECTIC DEPARTMENT,

### AND SPIRIT OF THE MEDICAL PERIODICAL PRESS.

---

*On Certain Cases of Intestinal Obstruction.*—Dr. James Patterson, in an interesting paper (*Glasgow Medical Journal*, July, 1855), makes the following useful practical remarks on this subject:

"In ordinary circumstances, the normal action of the bowels is sufficiently provided for by the stimulant and lubricating effects of the bile and intestinal secretions, which have been poured into the canal to aid the digestive process; but where the relationship of the food on the one hand, and of the digestive juices on the other, has been disturbed, the function of the bowels becomes altered in proportion. In some instances, the character of the ingesta may be such as to provoke a superabundant flow of the digestive secretions, when a diarrhœa may result, and prove as effective in removing the evil as it is salutary. But in the majority of instances, the opposite evil occurs. The secretions are thrown into the canal in quantities too small to insure the passage onward of matters losing their own moisture and becoming excrementitious. Besides, being too dry in character, the food is, in all likelihood, such as, little by little, to overstimulate the liver, until it has become congested and sluggish, with a diminished excretion of bile. The portal system is necessarily too much loaded, and the reäction being traced backwards, is found to insure a diminution of the secretive action of the intestinal follicles. It is unnecessary here to allude to the sedentary habits, and other well known external causes, so potent in inducing and perpetuating the condition of constipation; my present purpose will be fully served by calling attention to the internal changes, irrespective of the special causes which may have been at work in different individuals. In a majority of the cases in which the changes named occur, the different steps of the process of deviation from sound health are so insignificant, that, for a long time, they are unobserved; but, though delayed, the ultimate results are not less certain. For a time — it may be for many months—the bowels are daily evacuated, and the individual believes that their functions are perfectly performed, the trifling dryness of the matters excreted not being such as to attract his observation. All the while, however, the feces have been accumulating, but so gradually as to have given rise to no disturbance. The evacuation of to day contains part of what ought to have come yesterday, and so on, until the accumulation has become so considerable as to occasion the painful spasmodic attacks known as colic. In ordinary circumstances, a smart dose of purgative medicine and an enema are probably sufficient to remove the accumulation and to relieve the symptoms, and after a day or two the patient is as well as ever. Thus far the cases are sufficiently common in their occurence, and have been often d scribed with much minuteness; but those which follow present characters deserving of careful and special notice, and which — except in the place above noticed — have been overlooked amidst vague generalities.

"If, from the accumulation of residual matters being yet more gradual than usual, the acute symptoms have been delayed until the small intestine has its share of the load, the character of the symptoms is more urgent. The smallness of the opening leading from the ileum into the caput cæcum will explain how readily obstruction can occur there, even when the feces are but little removed from their natural consistence. To produce this obstruction we require nothing more than that the cæcum should be somewhat loaded, and that the whole colon should be deficient in its contractility from long-continued distension. I may here remark, that I have never met with any case, having the more special characters, without obtaining a history of previous attacks of what seemed to be ordinary colic. These accumulations, then, on the one side in the cæcum, and on the other in the ileum, retard, little by little, the current in the already sluggish portal circulation, and one result is serous effusion into the sub-mucous tissue; chemical changes in the retaining fecal matters occasion the evolution of various gases, which go to increase the distension; and, if continued long enough, these various sources of irritation will lead to inflammation of the parts. In most instances, however, the acute symptoms appear some time before there is any tendency to enteritis, so that this condition may probably be usually the result of neglect. While the *origo mali* has been quietly forming and acquiring potency, there has been no feeling of impending illness; a certain amount of distension may be present in the righth iliac region, and yet the individual feels so well that its presence never costs him a moment's reflection. The appetite may be, nay, usually is, excellent, and when the symptoms supervene suddenly, as usual, great astonishment is expressed that any pain should occur while the system is in such perfect health. The attack is precisely like that of ordinary colic, but is much more sudden and greatly more acute. The pain often appears to be excruciating, and remits but little. If asked to particularize the spot where the pain is chiefly felt, the patient will say that it is all over the abdomen, but examination shows that it is most severe in the right iliac region, and that, during the slight remissions, it is almost entirely restricted to this spot. The condition of the sufferer is sufficiently miserable; he can procure no sleep; there is generally sickness with vomiting of bilious matters; the tongue is swollen and indented, and is covered with a thin yellow fur; there is no appetite, and but little thirst; the urine is dark-colored and thick, but may be unaltered; the bowels are generally reported to have been quite regular, or only slightly constipated. Amid all the pain, the pulse is calm and regular, and but little, if at all, quickened. On examination of the abdomen, a distinct fullness is felt in the right iliac fossa. The rounded form of the intestines can be felt, firm at the lower part of the swelling, but becoming softer in a direction radiating from this point. Continuous pressure displaces flatulence, and diminishes the size of the swelling, but it speedily resumes its original bulk when the pressure is withdrawn. The tumor is the seat of a constant pain, which is generally very acute, and there are frequent paroxysms of pain stretching upward and across the abdomen. It is seldom that the comparatively local seat of the pain can be ascertained without a considerable amount of careful questioning, the frequent recurrence of the paroxysms having the effect of causing a belief in the mind of the patient that the pain is general.

Such is an outline of the more common symptoms attendant upon this distressing malady.

"In commencing the treatment, it is often indispensable to allay the acute pain before trying to procure evacuation of the loaded bowels. Purgatives given at first, in such cases, often do no more than aggravate the symptoms. I have found an enema, with tincture of assafœtida and solution of morphia in thin gruel, to produce the happiest effect, being followed by a refreshing sleep of several hours' duration. In such circumstances, we must not be deceived as to the patient's condition, for he will declare himself quite well, and if the bowels have acted in the interval, we may too readily believe him; but we may still feel the swelling at the side of the abdomen, and may readily produce severe pain by even moderate pressure. The symptoms have been merely lulled, but will assuredly return with even greater violence, if the treatment be suspended. The curative treatment is probably best commenced by the injection of gruel in large quantities, by means of the long tube recommended by Dr. O'Beirne. After some hours, there may be passed some mixed scybalæ, with the effect of lessening the swelling and partially subduing the tenderness at the right side. The injection may now be repeated in the same manner, and the result may be the discharge, after some time, of a still larger amount of softish scybalæ. At this stage, we may properly administer purgatives by the mouth, and note carefully their effect. If they produce free discharges, and still further diminish the swelling, the symptoms will probably subside rapidly; but if the swelling enlarges, and there is much movement of flatulence from one part to another, while, at the same time, there be little or no evacuation, we have quitted the injections too early, and must again have recourse to them. In many of the less severe cases, I believe much benefit may result from beginning with mild purgatives at once, employing, at the same time, the common enema apparatus. A few drops of laudanum may be beneficially added to each dose of the aperient.

"The purgative that I have been in the habit of employing is the Epsom salts, which seems, when largely diluted, and combined with ten or fifteen drops of the diluted sulphuric acid, to answer the object in view better than most other cathartics. The dose should, generally, not exceed two drachms, dissolved in twelve or sixteen ounces of water, and may be repeated every alternate hour until the effect be produced. Given in this way, and especially if the patient be desired to drink freely of gruel during its use, the sulphate of magnesia seldom fails to occasion free watery discharges, an effect of great value, when it is desired to clear the intestines of scybalous matter. Besides being more effective, saline purgatives appear to cause much less abdominal pain and sickness, if the precaution to dilute largely have been observed. The time during which the purgatives and the enemata are to be continued must be regulated by the need which calls for their use; but, on the whole, it is better not to give much medicine by the mouth, but rather to continue the injections, until their purpose is sufficiently accomplished. One reason for this precaution is, that even when free evacuations have been repeatedly obtained, and we believe the canal to be quite free, there will still remain an amount of tenderness on pressure, easily aggravated by whatever may tend to irritate. For this condition a fly-blister is generally required, and has the effect of reducing the remaining fullness, and of removing the tenderness. Sinapisms do not seem to answer, at least there has not been much benefit derived from their use in my hands. Curiously enough, the bowels very often discharge freely large quantities of thin feculent matter, several hours after the application of the blister. The diet all the while should be such as to support the strergth, and yet must be light and easy of digestion. With this object, beef tea, with bread, rice, sago, &c., may be given in small quantities, frequently repeated.

"These means, varied according to circumstances, form the essentials in the treatment of such cases, and, if commenced early enough, prove eminently successful. But where, on the contrary, delay has occurred until the parts have become inflamed, not only may the treatment of the obstruction require modification, but, in addition, the means calculated to subdue the inflammatory action must form a sequel to the steps already named. In cases of the latter description, the greatest care will be demanded, lest the obstruction should escape notice, for instances are not wanting where this has happened, the attention of the physician being directed solely to the obtrusively prominent symptoms of the enteritis. Symptoms of inflammatory action, with great abdominal tenderness, confined principally to the right side, may, with justice, raise a suspicion of obstruction in that portion of the intestinal canal. It is to this class of cases that the name *typhilitis* properly applies."

